# Fluorescence Detection of Cancer Stem Cell Markers Using a Sensitive Nano-Aptamer Sensor

**DOI:** 10.3389/fchem.2022.920123

**Published:** 2022-06-22

**Authors:** Jie Ding, Weiqiang Xu, Jing Tan, Zhifang Liu, Guoliang Huang, Shoushan Wang, Zhiwei He

**Affiliations:** ^1^ Guangdong Provincial Key Laboratory of Medical Molecular Diagnostics, The First Dongguan Affiliated Hospital, Guangdong Medical University, Dongguan, China; ^2^ Key Laboratory for Epigenetics of Dongguan City, China-America Cancer Research Institute, Guangdong Medical University, Dongguan, China; ^3^ Guangdong Engineering and Technology Research Center for Advanced Nanomaterials, School of Environment and Civil Engineering, Dongguan University of Technology, Dongguan, China; ^4^ The First Dongguan Affiliated Hospital, School of Basic Medical Science, Guangdong Medical University, Dongguan, China

**Keywords:** cancer stem cell, CD133, aptamer sensor, CdSe/ZnS QDs, AuNPs

## Abstract

Antigen CD133 is a glycoprotein present on the surface of cancer stem cells (CSCs), which is a key molecule to regulate the fate of stem cells and a functional marker of stem cells. Herein, a novel fluorescence “turn-on” nano-aptamer sensor for quantifying CD133 was designed using hybridization between CD133-targeted aptamers and partially complementary paired RNA (ssRNA), which were modified on the surface of quantum dots (QDs) and gold nanoparticles (AuNPs), respectively. Owing to the hybridization of aptamers and ssRNA, the distance between QDs and AuNPs was shortened, which caused fluorescence resonance energy transfer (FRET) between them, and the florescence of QDs was quenched by AuNPs. When CD133 competitively replaced ssRNA and was bound to aptamers, AuNPs-ssRNA could be released, which led to a recovery of fluorescent signals of QDs. The increase in the relative value of fluorescence intensity was investigated to linearly correlate with the CD133 concentration in the range of 0–1.539 μM, and the detection limit was 6.99 nM. In confocal images of A549 cells, the CD133 aptamer sensor was further proved applicable in lung cancer cell samples with specificity, precision, and accuracy. Compared with complicated methods, this study provided a fresh approach to develop a highly sensitive and selective detection sensor for CSC markers.

## Introduction

Cancer stem cells (CSC) refer to cancer cells with stem cell properties, which have the ability of self-renewal and multi-cell differentiation (Dick, 2008; Batlle and Clevers, 2017). CSCs are considered to have the potential to form tumors that develop into cancer, with it specifically being the source of formation of other cancer lesions during cancer metastases (Fidler, 2003; Kreso and Dick, 2014). In functional experiments, CSCs are defined as self-renewal cells and a source of formation of tumors when transplanted to immunodeficient mice (Lapidot et al., 1994). If CSCs are not completely eliminated in the treatment of cancer, cancer is easy to relapse and metastasize (Vlashi and Pajonk, 2015). Therefore, CSCs must be accurately detected and eradicated in the process of cancer treatment (Baum et al., 1992). Currently, multiple specific CSC-related markers have been identified and used to distinguish CSCs from the bulk of tumor cells (Hilbe et al., 2004; Phillips et al., 2006). The first marker was antigen CD133 (also known as promini-1), which was a CSC surface transmembrane glycoprotein that contained five transmembrane segments, two large extracellular loops, and two small intracellular loops (Singh et al., 2004; Miki et al., 2007). The protein is now widely adopted for the detection and isolation of CSCs in several cancers, such as breast cancer (Al-Hajj et al., 2003), brain cancer (Jin et al., 2010), lung cancer (Wu and Wu, 2009), and liver cancer (Suetsugu et al., 2006). In the past, the detection of the CD133^pos^ (CD133 overexpression) CSC had mostly relied on using immunehistochemical methods and flow cytometry, which required the participation of antibodies for accurate identification of CD133 (Kemper et al., 2010; Hermansen et al., 2011). Nevertheless, protein structure analysis showed that multiple N-glycan structures in CD133 could influence antibody binding due to their high sensitivity to glycosylation modification (Bidlingmaier et al., 2008). For overcoming the limitations of antibodies in the identification of CD133, researchers screened CD133-targeted aptamers by systematic evolution of ligands by exponential enrichment (SELEX) to substitute antibodies (Shigdar et al., 2013; Alibolandi et al., 2018). Aptamers are small single-stranded RNA or DNA oligonucleotides (∼20–60 nucleotides) that are easier to bind to targets and have the advantages of non-immunogenicity, low cost, high specificity, and strong affinity (Lakhin et al., 2013). In order to obtain a sensitive detection signal, aptamers were usually combined with fluorescence materials to form aptasensors or aptamer probes, which could provide powerful tools for the detection of targets.

The design mechanism of many fluorescence aptasensors utilized the FRET (Förster resonance energy transfer) principle, which is a photophysical process of non-radiative energy transfer between an excited donor and an acceptor by long-range dipole–dipole interactions (Xu et al., 2014). The efficiency of FRET is primarily dependent on the spectral overlap between the donor emission and acceptor absorption spectra. The effective distance between the donor and the acceptor in FRET is of order of 1–10 nm (Liu et al., 2011). In the past, organic dyes as the energy donor or the acceptor were the major components of FRET sensors. However, these sensors suffered some drawbacks, such as low resistance to photo-bleaching and chemicals, spectral cross-talk, and small Stokes shifts, which might cause wrong transmission signals and a high detection limit. With development of research, it has been shown that nanostructured particles could be particularly suited for FRET systems due to the high efficiency of energy transfer (Jennings et al., 2006; Rakshit et al., 2017). Quantum dots (QDs), also known as nanocrystals, are a kind of nanoparticles composed of II–VI or III–V elements, which have abundant energy electrons and holes limited by quantum (Oh et al., 2005). The continuous energy band structure becomes a discrete energy level structure with molecular characteristics, which can emit excellent fluorescence after excitation (Huang and Ren, 2012). Based on well-known advantages, QDs had been extensively exploited as energy donors for FRET systems (Chang et al., 2017; Wang et al., 2017). As an energy receptor frequently paired with QDs in FRET, gold nanoparticles (AuNPs) have unique physical and chemical properties, mainly including the broad absorption spectra in the visible region, good biocompatibility, easy surface modification, etc. (Ling and Huang, 2010; Quintiliani et al., 2014). In addition, AuNPs are ready for labeling by RNA via the sulfhydryl group (Nie and Emory, 1997). The FRET sensor system constructed by QDs and AuNPs could be divided into two types according to the quenching or recovery of fluorescence: the “turn-off” mode and the “turn-on” mode. In the past, most of the fluorescence nanosensors were designed with the “turn-off” mode, where many factors could influence the detection effect and detection limit (Xia et al., 2012). By comparison, the fluorescence “turn-on” nanosensors employed in FRET-based systems could reduce the probability of false positives (Wang et al., 2012). Therefore, in this article, a nano-aptamer sensor composed of classical CdSe/ZnS QDs and AuNPs with a size of 25 nm was designed to detect CD133 in the fluorescent “turn-on” mode.

**SCHEME 1:**
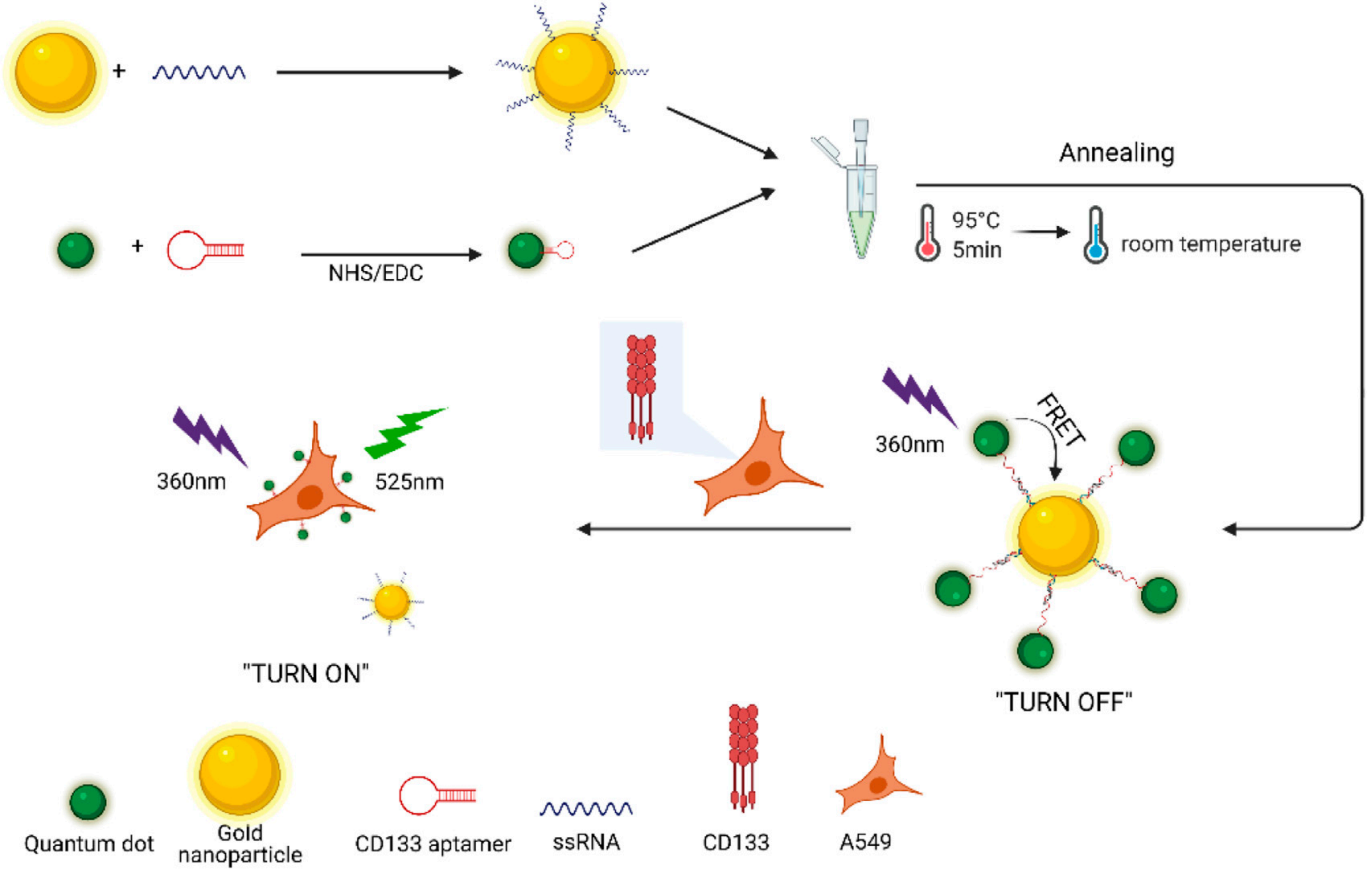
Fluorescence detection of CD133 based on the “turn-on” FRET nano-aptamer sensor.

In this study, a novel “turn-on” FRET nano-aptamer sensor with CdSe/ZnS QDs and AuNPs as the energy donor–acceptor pairs was developed for detecting CD133, a CSC-related marker. Initially, the CD133-targeted aptamer and partially complementary paired RNA (ssRNA) were screened based on the sequence specific for CD133 and conjugated to QDs and AuNPs, respectively. As shown in Scheme 1, FRET occurred when the CD133 aptamer was hybridized with ssRNA, allowing one to bring QDs and AuNPs into close proximity; then the fluorescence of QDs was quenched by AuNPs. In principle, the fluorescence recovery of QDs was related to the ability of CD133 to competitively replace ssRNA and bind it to the CD133-targeted aptamer. Based on the standard curve obtained by fluorescence recovery of QDs, the detection limit (LOD) of the nano-aptamer sensor was calculated to be around 6.99 nM for CD133 detection. Meanwhile, the specificity, precision, and accuracy of the nano-aptamer sensor were examined, and its applicability in lung cancer cell samples was also validated. It was believed that this simple “turn-on” FRET nano-aptamer sensor would offer a promising approach for CSC marker detection with a low LOD and good selectivity.

## Results and Discussion

### Synthesis and Characterization of AuNPs-ssRNA

**FIGURE 1:**
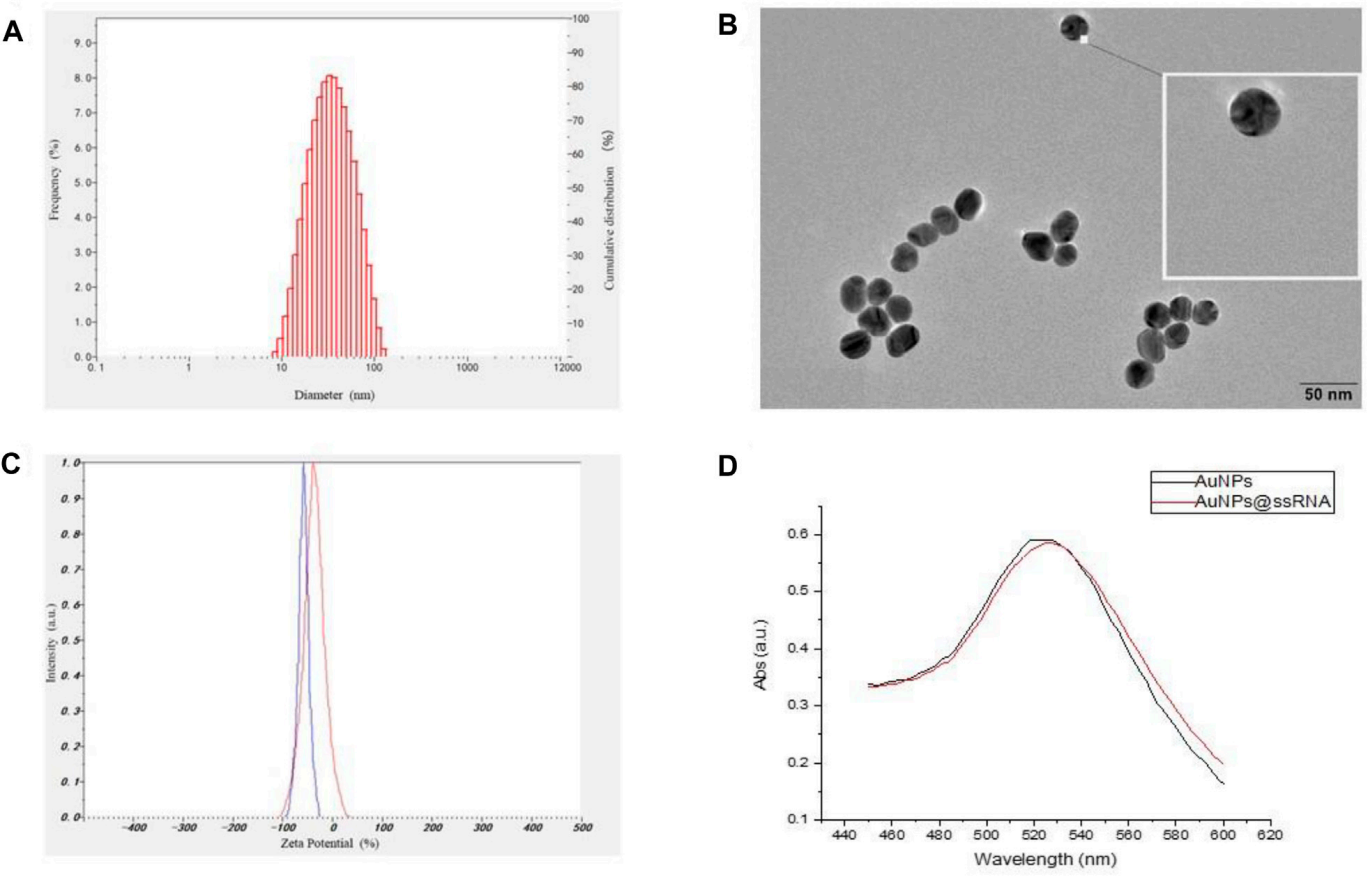
**(A)** The hydrodynamic size distribution of AuNPs by DLS measurement. **(B)** TEM image of AuNPs. **(C)** The zeta potentials of AuNPs and AuNPs-ssRNA. **(D)** The UV–Vis spectra of the AuNPs and AuNPs-ssRNA.

The synthetic approach of the fluorescence “turn-on” nano-aptamer sensor is summarized in Scheme 1. First, AuNPs with a size of 25 nm were synthesized according to the method reported by Wang et al. (2019). The dynamic light scattering (DLS) results showed that AuNPs were well-dispersed with an average hydrodynamic size of 30 nm (Figure 1A). At the same time, the TEM image of AuNPs is exhibited in Figure 1B, where AuNPs are of a round shape and well-dispersed. Subsequently, the ssRNA with the endpoint modified sulfhydryl group was directly bound to the surface of AuNPs through coordination bonds. After the modification of ssRNA, the zeta potential of AuNPs dramatically increased from −58 mV to −36 mV (Figure 1D), indicating the successful modification of ssRNA on AuNPs. In Figure 1D, the UV–Vis spectra of AuNPs and AuNPs-ssRNA showed that the adsorption peak of AuNPs was at 523 nm while that of AuNPs-ssRNA was at 525 nm. The red-shift in the adsorption spectra further demonstrated that ssRNA was successfully conjugated onto the AuNPs’ surface. Since RNA hybridization required annealing, agglomeration would occur in subsequent experiments if the concentration ratio of AuNPs to ssRNA was greater than or less than 1:200 (molar concentration ratio). Thus, the optimum concentration ratio of AuNPs to ssRNA was chosen to be 1:200.

### Synthesis and Characterization of QD-Aptamers

**FIGURE 2:**
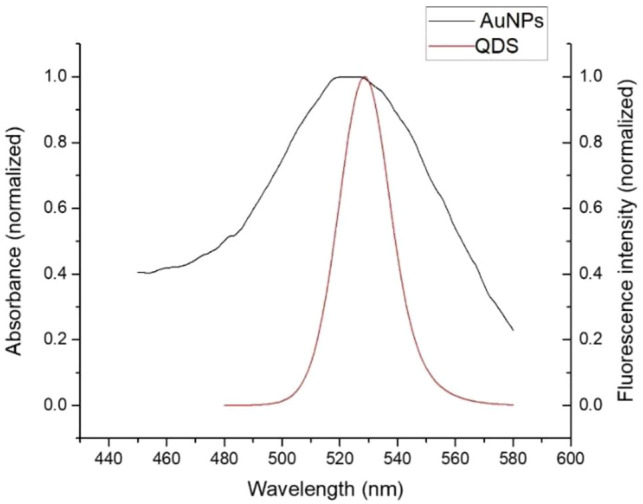
After normalization, the QDs’ fluorescence peak overlapped with the AuNPs’ absorption peak.

**FIGURE 3:**
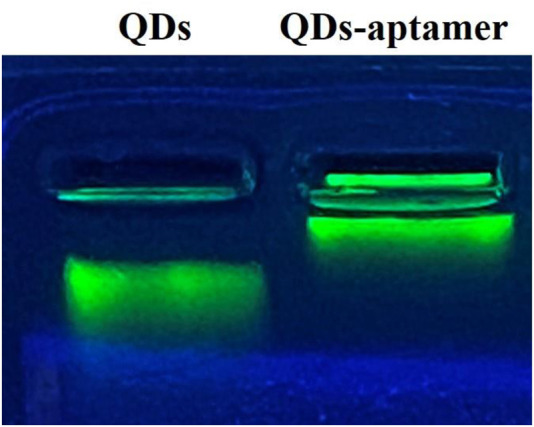
The agarose gel electrophoresis of QDs and QD-aptamers showed that QDs without a conjugated aptamer ran faster in agarose gel.

The fluorescence spectra in Figure 2 showed that the emission peak was 525 nm for CdSe/ZnS QDs; there was an overlap between the emission spectrum of QDs and the absorption spectrum of AuNPs around 525 nm, indicating that the QDs and AuNPs could work as a FRET donor–acceptor pair. Then, the CD133-targeted aptamer was conjugated onto CdSe/ZnS QDs by an ammonia carboxylation reaction, and the ratio between aptamer and QDs was optimized. Due to the large surface area of QDs, there were multiple binding sites on the surface of QDs to couple with aptamers. After the overdose aptamer reacted with QDs, the unbound aptamer was removed using an ultrafiltration tube. For proving the aptamer conjugation, QDs and QD-aptamers were monitored by agarose gel electrophoresis (Figure 3), which implied that the conjugation efficiency reached a plateau when the aptamer concentration exceeded 7 times the molar concentration of QDs. Herein, 7:1 was chosen as the optimal concentration ratio of aptamer-bound QDs.

### Synthesis and Characterization of Fluorescence “Turn-On” Nano-Aptamer Sensors

**FIGURE 4:**
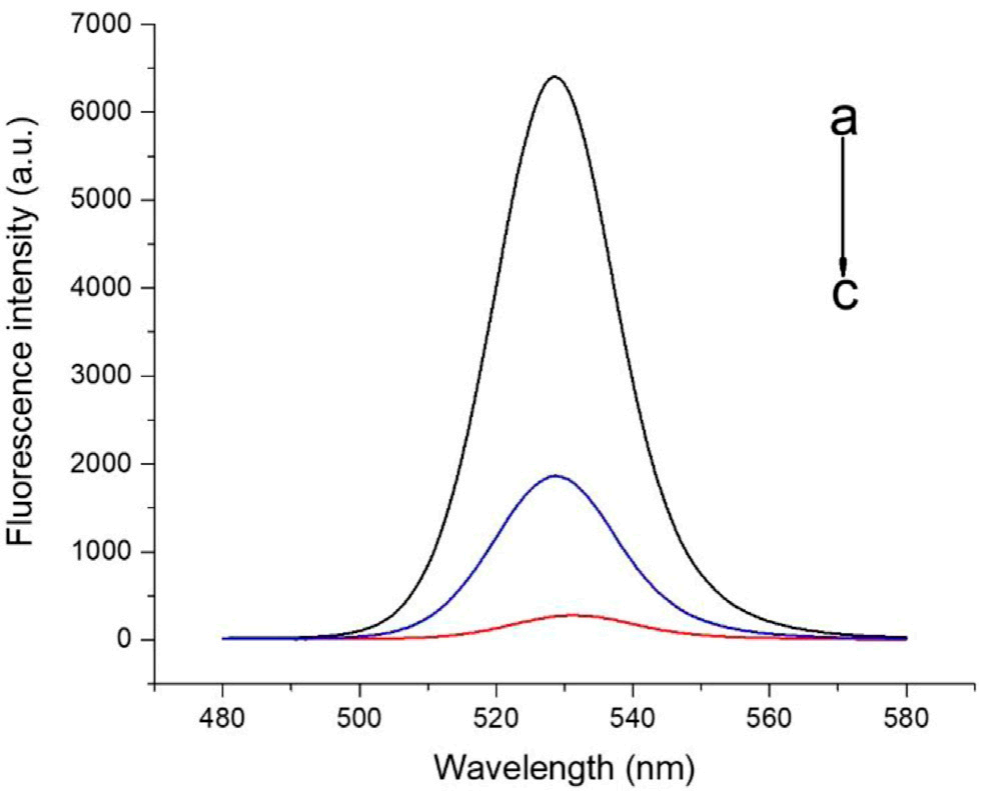
The fluorescence spectra of a) QD-aptamers, b) the mixture of QD-aptamers and AuNPs, and c) the hybridization of QD-aptamers and AuNPs-ssRNA (aptamer sensor).

**FIGURE 5:**
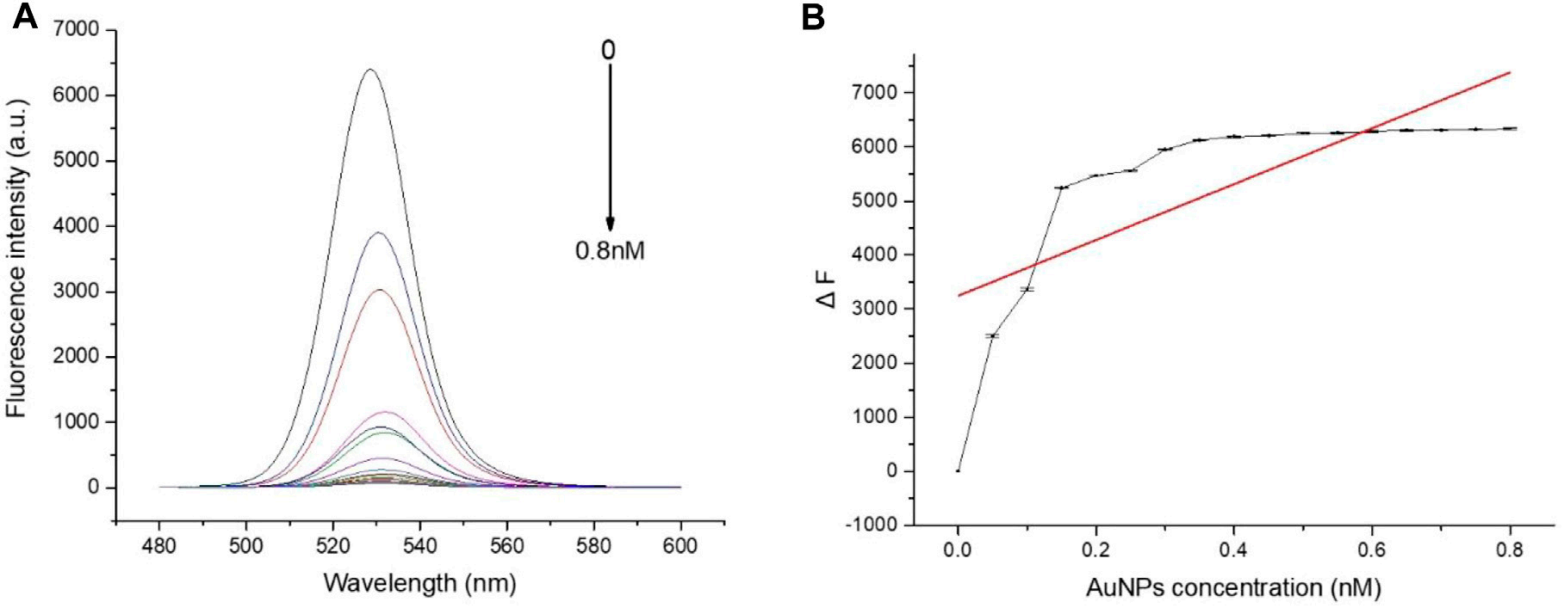
**(A)** The fluorescence spectra of QD-aptamers hybridized with different concentrations of AuNPs-ssRNA; **(B)** When the hybridization concentration ratio of QD-aptamers to AuNPs-ssRNA was 10:1, the fluorescence intensity of QDs reached the plateau stage. The aptamer sensor for the detection of targeted CSC markers.

To evaluate whether FRET occurred in our design, these fluorescence spectra were measured, including QD-aptamers, the mixture of QD-aptamer and AuNPs, and the hybridization of QD-aptamers and AuNPs-ssRNA (aptamer sensor) (Figure 4). The fluorescence spectrum for free QDs showed a maximum peak at 525 nm, which was close to the maximum of absorption measured for AuNPs. It suggested that CdSe/ZnS QDs and AuNPs could serve as a suitable donor–acceptor pair. It could also be seen that the fluorescence band (the full width at half-maximum fluorescence intensity, FWHM) was relative narrow and symmetric, which suggested that both QDs and the aptamer sensor were homogenous and monodisperse. According to the past studies, FRET could operate when an energy donor to an acceptor was in close proximity (≤10 nm). The chain length of the CD133-targeted aptamer indicated that the distance between the donor and the acceptor was definitely achieved in the studied system. As could be seen in Figure 4C, when the hybridization between QD-aptamers and AuNPs-ssRNA is driven by the specific pairing of aptamers and partial complementary ssRNA, the fluorescence signal of QDs was quenched dramatically by AuNPs due to the occurrence of FRET. However, when there was no ssRNA conjugated to AuNPs (Figure 4B), the hybridization was not in operation, and the fluorescence intensity of QDs was only partially reduced. The spectra results indicated that the efficiency of FRET was significantly improved by linking QDs and AuNPs through aptamer–ssRNA hybridization. To ensure adequate hybridization between the aptamer and ssRNA, the hybridization time was fixed at 90 min (annealing time). As shown in Figure 5A, the fluorescence signal intensity of QDs decreased with the increase in AuNPs and reached quench equilibrium when the molar concentration ratio of QDs to AuNPs was 10:1 (Figure 5B). Finally, the synthesis conditions of the aptamer sensor were determined where the hybridization reaction time was 90 min and the concentration ratio was 10:1.

**FIGURE 6:**
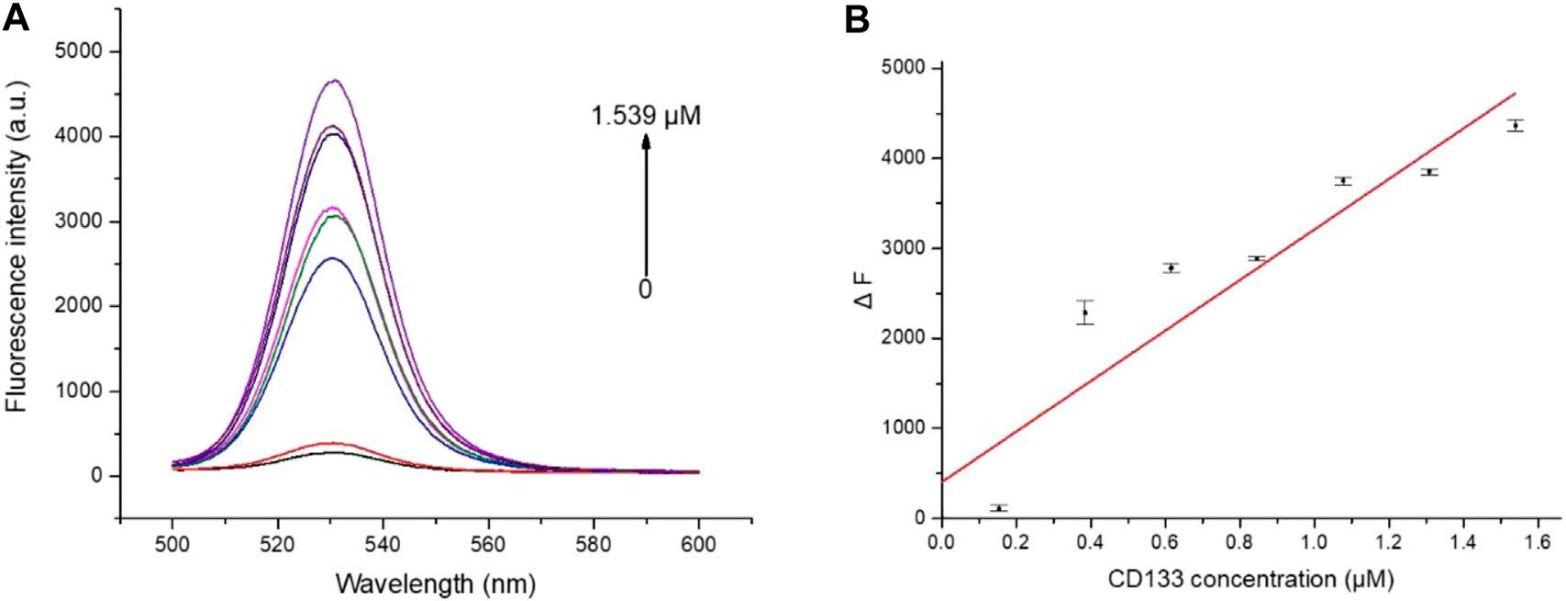
**(A)** The fluorescence spectra of the aptamer sensor treated with different concentrations of CD133. **(B)** The plot of ΔF as a function of CD133 concentration.

To explore the fluorescence “turn-on” efficiency of this FRET aptamer sensor, a series of concentrations of CD133 were tested from 0 to 1.539 μM. Herein, a fixed number of QD-aptamers (5 nM) was used. As a result of competitive replacement of ssRNA by CD133, the distance between QDs and AuNPs increased significantly, and fluorescence recovery was realized. The recovery efficiency was expressed by ΔF = F_C_ − F_0_, where Fc is the fluorescence intensity of QDs after recovery and F_0_ is the original fluorescence intensity of the aptamer sensor. As shown in Figure 6A, the recovery efficiency gradually increased with the increase in CD133 and reached a maximum of 68% with 1.539 μM CD133, and then the recovery efficiency was almost unchanged when the concentration of CD133 further increased. Figure 6B indicated the fluorescence signal recovery (F_C_ − F_0_) versus a series of CD133 concentrations. Then the detection limit (LOD) was determined by the standard curve, which was around 6.99 nM.

### The Specificity of the Aptamer Sensor

**FIGURE 7:**
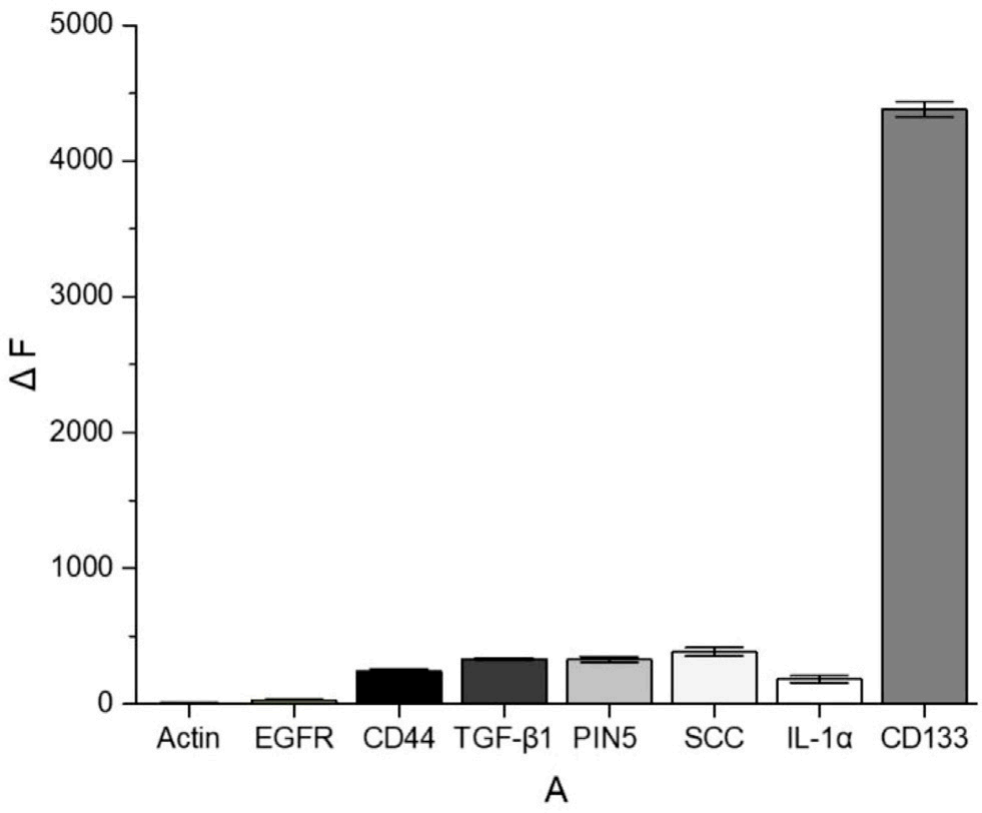
ΔF comparison between CD133 and other common cancer-associated proteins.

To evaluate the specificity, the aptamer sensor was treated with multiple common cancer-associated proteins and compared with CD133. All the experiments used the same concentration (2 μM) of proteins for comparison. As shown in Figure 7, these interfering proteins hardly recovered the fluorescence of QDs. For example, the recovery efficiency of CD133 was around 68%, while that of SCC was merely around 5%. The results indicated that this FRET “turn-on” nano-aptamer sensor exhibited high selectivity.

### *In Vitro* Imaging of Cell Response to the Aptamer Sensor

**FIGURE 8:**
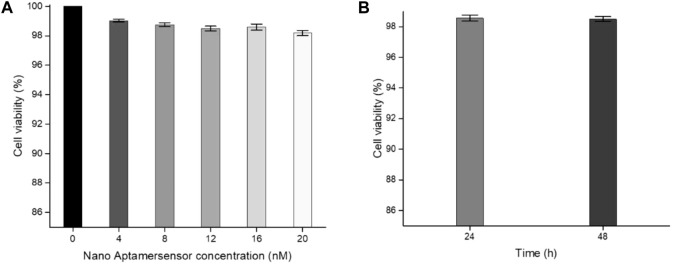
**(A)** Cell viability of A549 cells incubated with different concentrations of the aptamer sensor for 24 h. **(B)** Cell viability of A549 cells incubated with a 20 nM aptamer sensor for 24 or 48 h.

**FIGURE 9:**
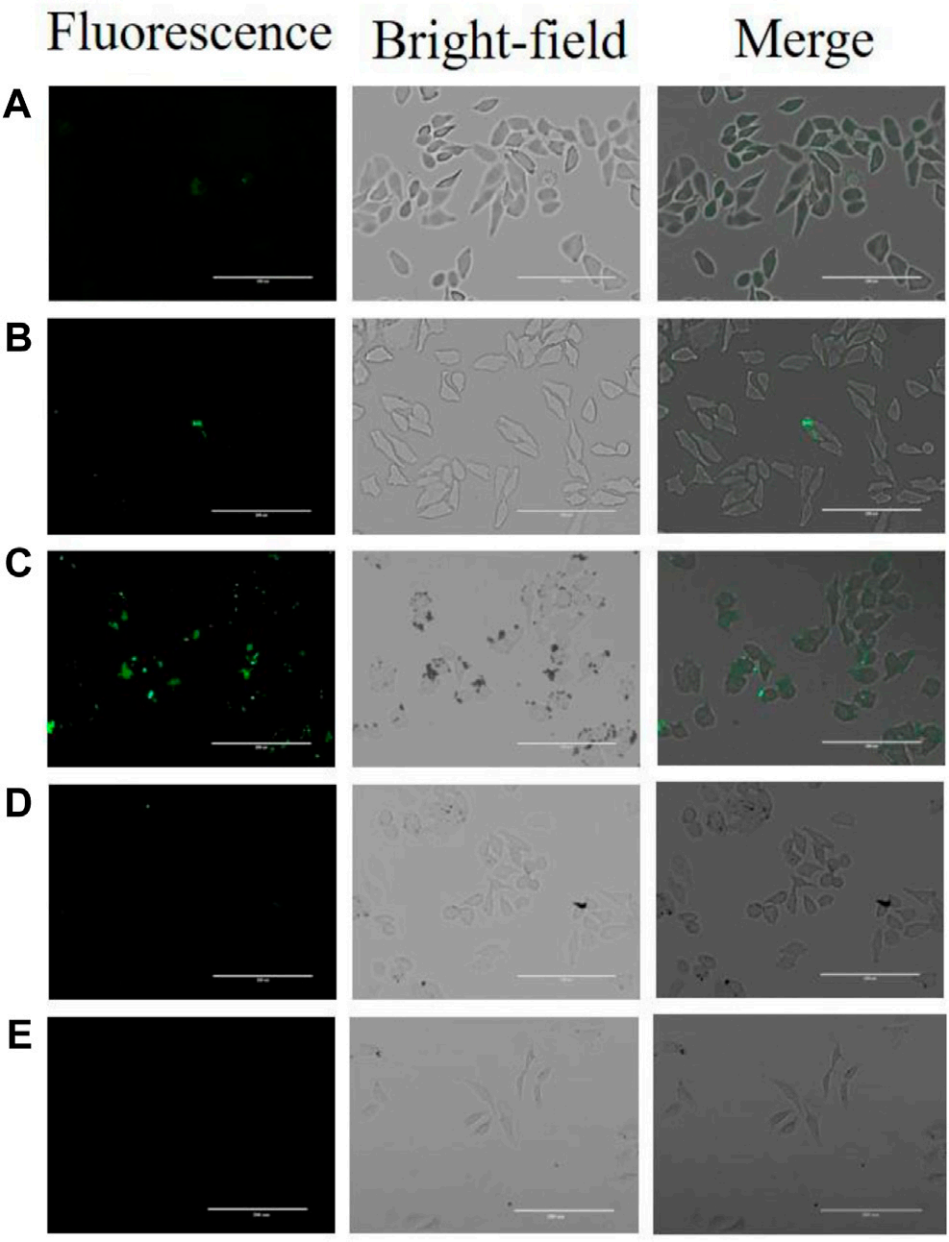
Confocal microscopy images of A549 cells or HuH-7 cells following treatment of **(A)** QDs, **(B)** QD-aptamers, **(C)** aptamer sensors, and **(D)** pretreated cells with CD133-targeted aptamers for 24 h and then incubated with the aptamer sensor. **(E)** HuH-7 cells were treated with the aptamer sensor.

In order to study the biocompatibility of the nano-aptamer sensor, CCK-8 assays were used to evaluate the cytotoxicity of the aptamer sensor. The results indicated that A549 cells still maintained high viability after incubating for 24 h with the aptamer sensor up to 20 nM (Figure 8A). Even if the incubation time was prolonged to 48 h, it would not affect the cell viability (Figure 8B). In other words, if the concentration of the aptamer sensor was not more than 20 nM, then the aptamer sensor had low toxicity to cells and could be used for cellular imaging. Furthermore, to demonstrate the capacity of the fluorescence “turn-on” FRET aptamer sensor in selectively detecting CD133^pos^ cells, the lung cancer cell A549 (CD133^pos^ cell) was treated with the aptamer sensor. After incubation with QD-aptamers or the aptamer sensor for 8 h, strong fluorescence signals were detected from A549 cells by confocal microscopy imaging (Figures 9B,C), while the control experiment using QDs without the aptamer at the same concentration showed weak fluorescence (Figure 9A). For verifying that this “turn-on” phenomenon was ascribed to the competitive release of AuNPs-ssRNA, the binding site of A549 cells was saturated with the CD133-targeted aptamer for 24 h before incubation with the aptamer sensor. It turned out that the fluorescence signal from A549 cells was negligible, as shown in Figure 9D. A negative control experiment performed with CD133-negative HuH-7 human hepatoma cells showed that the fluorescence recovery of the aptamer sensor was not observed (Figure 9E), further indicating that the release of AuNPs-ssRNA was from the specific binding between the aptamer and CD133.

## Experimental Section

### Materials and Equipment

Chloroauric acid (HAuCl_4_) and sodium citrate were purchased from Shanghai Aladdin Biochemical Technology Co. Ltd. Carboxyl-modified quantum dots (-COOH QDs) were obtained from Suzhou Xingshuo Nanotechnology Co. Ltd. 1-ethyl-(3-dimethylaminopropyl) carboimide (EDC) and N-hydroxysuccinimide (NHS) were purchased from Shanghai Macklin Biochemical Co. Ltd. The amino-modified CD133 aptamer (5′-NH_2_-CCCUCCUACAUAGGG-3′) and CD133 aptamer partial complementary pairing part (ssRNA, 5′-SH-CCCUAUG-3′) were synthesized by Shanghai Generay Biotech Co. Ltd. All buffers were prepared with ultrapure water, which was purified to a resistivity of 18.2 MΩ*cm by a milli-Q system (Merck Millipore, United States). A dynamic light scattering particle size analyzer (SZ-100, Horiba, Japan) was used for measuring the zeta potential and particle size. The morphology of nanoparticles was characterized by transmission electron microscopy (JEOL JEM-2100 F, JEOL Ltd., Japan). The UV–Vis absorption spectra were recorded on a microspectrophotometer (NanoDrop ND-1000, Thermo Scientific, United States). Fluorescence spectra were obtained using a fluorescence spectrophotometer (F-7000, Hitachi, Japan). Fluorescence images were recorded using a confocal laser scanning microscope (TCS SP8 Leica, United States).

### The Synthesis of Gold Nanoparticles

All reaction instruments needed to be soaked in aqua regia or concentrated alkali and washed with ultrapure water 3 to 4 times. Then 250 μl of 0.4 M chloroauric acid (HAuCl_4_) and 50 ml of ultrapure water were added to a round bottom flask, continuously stirred, and heated to boiling. After boiling, 10 ml of 38 mM sodium citrate solution was quickly injected, and then the solution was heated to boiling again. The color of the solution gradually changed from light yellow to wine red; the solution was boiled for another 15 min until the reaction was complete. After cooling, it was filtered by an ultrafiltration membrane and stored at 4°C.

### AuNP-Coupled Nucleotide Single Strand (AuNPs-ssRNA)

The prepared AuNPs were incubated with a sulfhydryl nucleotide single chain (ssRNA) in a ratio of 1:200 for 24 h, and then 1 M sodium phosphate buffer (PBS, 1 M NaCl, 100 mM Na_2_HPO_4_ and NaH_2_PO_4_, pH = 7.4) was added step by step. At least for 30 min between each step, the final concentration of PBS reached 0.1 M. The solution was incubated at room temperature for 40 h. Finally, the excess ssRNA was removed by centrifugation (13,800 rpm, 20 min) and redispersed in 0.1 M PBS.

### The Characterization of AuNPs-ssRNA

Since coupling nucleotides with AuNPs would reduce the anions on their surface, the salt solution was added to resist the cations on the surface of AuNPs in order to prevent agglomeration and maintain stability, while uncoupled or incompletely coupled AuNPs would agglomerate and change color. Therefore, salt solution of the same concentration and volume could be added to the reaction mixture of AuNPs and ssRNA to observe whether it changes color and agglomerates, and the reaction degree between AuNPs and ssRNA is judged.

### QD-Coupled Aptamers (QD-Aptamers)

Carboxyl CdSe/ZnS QDs (40 μl, 0.6 nM) were activated with 60 μl (50 mM) EDC and 30 μl (25 mM) NHS under mild stirring for 15 min. The activated QDs were incubated with an amino aptamer at different mass ratios for 24 h, and then the unreacted aptamer was separated using an ultrafiltration centrifuge tube. The obtained QD-aptamers were redispersed in PBS.

### The Detection of Fluorescence Change After Hybridization Between QD-Aptamers and AuNPs-ssRNA

The obtained QD-aptamers were divided into two groups. One group was added with the stock solution of AuNPs, and the other was added with AuNPs-ssRNA. The nucleotide chains were complementary paired by annealing (adding the annealing buffer at 95°C for 5 min and gradually reducing to room temperature), and then the fluorescence value of the mixture was measured at the same time to verify that the strong fluorescence quenching effect was due to the complementary pairing of the nucleotide chains, shortening the distance between the energy donor and the acceptor. Based on the fluorescence change in QDs, the reaction time and the molar ratio of AuNPs to QDs were determined. The final fluorescence “turn-on” nano-aptamer sensor was obtained.

### Highly Selective Detection of CD133 by Aptamer Sensors

After the aptamer sensor reacted with CD133 and other interfering proteins (actin, EGFR, CD44, TGF-β1, PIN 5, SCC, and IL-1α), the fluorescence value of the reaction solution was measured to verify that CD133 could specifically bind to the CD133-targeted aptamer on the surface of QDs by replacing AuNPs-ssRNA. The aptamer did not respond to other interfering proteins, which proved its specificity.

### Fluorescence Imaging of CD133^pos^ CSCs Using the Aptamer Sensor

After passage, lung cancer cells A549 were inoculated into confocal dishes and divided into four groups for different experimental operations. The first group was incubated with single QDs for 8 h. The second group was incubated with QD-aptamers for 8 h. The third group was incubated with aptamer sensors for 8 h. The last group was incubated with a single aptamer for 24 h and then with the aptamer sensor for 8 h. The concentration of QDs in the four groups was the same. After incubation, the solution in the dishes was washed with PBS twice, and the fluorescence imaging of the cells was observed under a confocal laser scanning microscope. As control, HuH-7 cells were incubated with an aptamer sensor for 8 h to detect the fluorescence of QDs.

## Conclusion

In summary, a novel aptamer sensor was designed to detect CD133 that consisted of QDs modified with a CD133-targeted aptamer and AuNPs conjugated with partially complementary paired RNA (ssRNA). The hybridization between the aptamer and ssRNA brought QDs and AuNPs into close proximity to trigger FRET, where the change in fluorescence intensity of QDs was recorded using a fluorescence spectrophotometer. CD133, a CSC marker, competitively replaced ssRNA, resulting in the decrease in the FRET effect between QDs and AuNPs and the recovery of QD fluorescence. The experiments demonstrated the feasibility of this “turn-on” FRET nano-aptamer sensor for CD133 detection with an LOD of 6.99 nM. Moreover, the aptamer sensor was able to detect CD133 on the surface of A549 cells by displaying the fluorescence of QDs through confocal images. These results suggested that the aptamer sensor is a sensitive and reliable sensor for the detection of CD133 and offers a simple yet promising testing tool for CSC marker detection.

## Data Availability

The original contributions presented in the study are included in the article/Supplementary Material; further inquiries can be directed to the corresponding authors.
